# Exogenously applied *Casuarina equisetifolia* leaf extracts act as an osmoprotectant on proline accumulation under drought stress in local rice from Indonesia

**DOI:** 10.3389/fpls.2023.1210241

**Published:** 2023-08-03

**Authors:** Yustina Carolina Febrianti Salsinha, Dwi Setyo Rini, Didik Indradewa, Diah Rachmawati, Taufan Alam, Yekti Asih Purwestri

**Affiliations:** ^1^ Research Center for Genetic Engineering, National Research and Innovation Agency, Bogor, West Java, Indonesia; ^2^ Research Center for Biotechnology, Universitas Gadjah Mada, Yogyakarta, Indonesia; ^3^ Department of Agronomy, Faculty of Agriculture, Universitas Gadjah Mada, Yogyakarta, Indonesia; ^4^ Department of Tropical Biology, Faculty of Biology, Universitas Gadjah Mada, Yogyakarta, Indonesia

**Keywords:** *Casuarina equisetifolia*, proline accumulation, exogenous osmoprotectant, rice, drought stress

## Abstract

The effects of exogenously supplied osmoprotectants in crops have not yet been extensively studied. In this study, an osmoprotectant containing a high concentration of proline (2.5 g mol^−1^ FW) was obtained from a *Casuarina equisetifolia* leaf extract. The effect of the extract was evaluated in local Indonesian rice cultivars Boawae Seratus Malam (BSM), Gogo Jak (GJ), Situ Bagendit (SB) (drought-tolerant), Kisol Manggarai (KM) and Ciherang (drought-susceptible) cultivars under drought at the morphological, physiological, and genetic levels. Under drought, the KM showed an increased level of *OsWRKY*, *OsNAC*, *OsDREB1A*, and *OsDREB2A* expression after application of the osmoprotectant, leading to the activation of proline synthesis genes including *OsP5CS1*, *OsP5CR*, and *OsProDH*, while the tolerant cultivars (BSM, GJ, and SB) showed no difference. The content of chlorophyll, carotenoids, anthocyanins, ascorbate peroxidase, catalase, and superoxide dismutase activities also increased in GJ and KM, during drought stress and applied osmoprotectants, but remained low in the BSM. We conclude that the foliar application of osmoprotectants derived from *C.equisetifolia* caused an accumulation of proline in susceptible plants. The existence of these extracts stabilizes leaf cells and supports photosynthetic compartments and carbon assimilation in plants, leading to growth.

## Introduction

Rice (*Oryza sativa* L.) is the world’s largest food crop, mostly cultivated in Asia (Aslam et al., 2022). Increasing the global production of rice now and in the future has many challenges, including limited land and water availability due to drought (Dawe, 2013; Shahzad et al., 2020). There are several strategies to mitigate the threat of drought stress, such as screening drought-tolerant cultivars, designing water-efficient planting, and applying protectant compounds and biostimulants that can induce plant tolerance to drought (Bertolino et al., 2019; Upadhyay, 2019; Aslam et al., 2022).

Different ways of using plant extracts to prevent the effects of drought have been studied. Research by Pandey et al. (2016) showed that the application of an *Ocimum* extract increased the expression of the *Dehydrin* gene, while the expression of the *Aquaporin* gene was decreased in drought-stressed rice plants. Application of other plant extracts including kirinyuh leaves (*Chromolaena odorata*) has no significant effect on shoot length, fresh weight, or dry weight of rice seedlings, but affects the relative water content of rice seedlings (Wahyuni et al., 2018). Another study (Lalarukh et al., 2022) showed that organic extracts from *Moringa* leaves can induce immunity in plants under nutritional and drought stress to increase their survival. Providing plant extracts is one way to increase crop productivity in arid areas through organic methods, but the regulatory impact of these extracts as osmoprotectants on crop plants under drought has not been sufficiently elucidated.

Drought stress threatens plants in an extremely harmful way by causing abnormal growth, reducing water use efficiency, and disrupting physiological processes through restricted water transport (Kulkarni et al., 2017). Drought stress decreases turgescence and damages the osmotic balance of plants (Hassanein et al., 2021). To survive, plants have evolved various responses as adaptations to drought stress conditions. Adaptation strategies include the development of an extended root system (Kim and Lee, 2020), development of smaller and thicker leaves (Bertolino et al., 2019; Salsinha et al., 2021), increased diffusion resistance (Darmadi et al., 2021), reduced water loss through leaf wilting (Hassanein et al., 2021), and reduction in transpiration rate (Farooq et al., 2009; Aslam et al., 2022).

Drought tolerance is a complex property of plants that result from several genetics, morphological, biochemical, physical, and physiological adaptive traits (Aslam et al., 2022). At the transcriptional level, the regulatory response to drought stress involves members of several transcription factor (TF) families, such as WRKY (a 60-amino-acid region that is defined by the conserved amino acid sequence WRKYGQK at its N-terminal end), NAC (NAM, ATAF1/2, and CUC1/2), and AP2 (APETALA2)/ERF (ethylene-responsive factor) (Kulkarni et al., 2017; Wu et al., 2022). One AP2 subfamily is the DREBs (dehydration-responsive element-binding proteins) (Baillo et al., 2019; Herawati et al., 2021).

At the metabolic level, enzymatic and non-enzymatic oxidative reactions are mandatory for the protection against drought stress. Another protective approach for the osmotic adjustment of plants is the application of osmoprotectant (Chen et al., 2020). Organic compounds used as osmoprotectants are proline (Pro), glycine betaine (GB), and dissolved sugars (Cha-um et al., 2013). Their accumulation lowers the intracellular water potential. In addition, Pro also acts as a metabolic marker against drought stress (Islam et al., 2022), thereby stabilizing the intracellular and macromolecular structures of soluble and membrane-bound proteins in complexes during abiotic stress (Nounjan et al., 2012; Herawati et al., 2021; Shemi et al., 2021).

This protective effect Pro and GB in rice are also achieved by their exogenous supply to plants under drought stress (Hasanuzzaman et al., 2014; Shemi et al., 2021). The application of foliar osmoprotectants, such as polyamine, Pro, ABA, GB, GA3, sugar compounds, and salicylic acid, can increase turgor pressure and induce antioxidant activity against ROS (reactive oxygen species) (Chen et al., 2020; Aslam et al., 2022; Atteya et al., 2022).

Exogenous GABA application (Yong et al., 2017) activates 1-pyrroline-5-carboxylate synthetase (P5CS) activity, which leads to Pro accumulation and elevated proline dehydrogenase (PRODH) activity in periods of prolonged drought. Based on their increased antioxidant defense, plants under high-salinity stress revealed a better protective function for Pro than for GB (Hasanuzzaman et al., 2014). Regarding productivity, Farooq et al. (2008) showed that supplemented GB to plants potentially maintains optimal growth and even increases the productivity of rice plants. Foliar application of 100 mM GB also stabilizes plant metabolic processes and increases productivity (Jalal-ud-Din et al., 2015). The application of Pro and sucrose on rice grains also improved the flavor quality and nutrient content of rice (Arsa et al., 2016).

The molecular action of Pro as an osmoprotectant has not extensively been studied, particularly with respect to its roles as a free radical scavenger, growth promoter, enzyme activator, osmoprotectant in abiotic stress, and as a regulatory factor in other physiological processes (Pandey and Shukla, 2015; Hosseinifard et al., 2022). The objective of the present study was to analyze the specific impact of an exogenously applied Pro-rich leaf extract as a source of osmoprotectants for local rice cultivars in Indonesia. A crude leaf extract was derived from the halophyte plant *Casuarina equisetifolia*. We hypothesized that this plant extract with a higher Pro content would be an adequate source of osmoprotectants for the improved adaptation of local rice plants with increased growth rate and elevated resistance in drought. The effect of applied exogenous osmoprotectant could be observed at the expression level of genes related to the proline synthesis pathway as well as to other physiological processes downstream from the Pro accumulation.

## Materials and methods

### Materials used and research design

Five rice cultivars (**Table 1**) were used. Cultivation and treatment were carried out in a greenhouse of the Sawitsari Research Station (7°45’22” latitude, 110°23’18” longitude), Faculty of Biology, Universitas Gadjah Mada Yogyakarta, Indonesia with the air temperature ranging between 29–34°C (day) and 21–25°C (night) during January–June, photosynthetic photon flux density about 500 µmol m^−2^ s^−1^, air humidity >80%, and average rainfall 300–400 mm.

**Table 1:** Five rice (Oryza sativa, indica) cultivars (Salsinha et al., 2021).

No.	Cultivar	Abbreviation	Response to drought
1	Boawae Seratus Malam	BSM	Tolerant
2	Gogo Jak	GJ	Tolerant
3	Kisol	KM	Susceptible
4	Ciherang	CH	Susceptible (control)
5	Situ Bagendit	SB	Tolerant (control)

The research design was a randomized complete block design with three variables: cultivar (five cultivars), drought stress treatment (two levels), and exogenous osmoprotectant treatment (three concentrations) with three replications, consisting of one individual plant each. Each block of 60 cm × 100 cm contained 15 individual plants of different cultivars at random positions. The sowing period lasted until the plants were 21 days old, at which point they were planted and drought stress treatment was applied until the plants reached the final vegetative stage at 49 DAPs (days after planting); the foliar osmoprotectant treatment was carried out at 21 DAP.

### Drought stress treatment

The drought stress treatment followed the IRRI protocol (Serraj et al., 2008) by applying the FTSW (fraction of transpirable soil water). Before the FTSW treatment, the total transpiration of soil water (TTSW) of each different cultivar was analyzed. From the TTSW value, the exact weight of pot and plant at each FTSW was calculated. Two levels of FTSW were used: FTSW 1 for control and FTSW 0.2 for drought stress. The TTSW value was separately determined by calculating the difference of pot and plant weight at 100% field capacity (P0) with the weight of the pot and plant when the plant shows a permanent wilting point (Pi) character with Eq. 1 (Salsinha et al., 2022):


(1) TTSW=PO(g)−Pi(g)

(2) Wt (ml) = FTSW×TTSW

(3) Pt(g)=PO−(TTSW−Wt)

During the treatment, the amount of soil water (Wt) kept stable was calculated for each FTSW stage for each cultivar using Eq. 2. The weight of soil and pots (Pt) during the treatment period was kept stable using Eq. 3.

### Exogenous osmoprotectant treatment

Exogenous osmoprotectant was obtained from a crude leaf extract of *C. equisetifolia*. GB and Pro are highly concentrated compounds in *C. equisetifolia* leaf extract that can enter and accumulate in rice tissue and can act as an osmoprotectant (Basuki and Prajitno, 2014). The leaves were sampled from the coastal area of Gunung Kidul, D.I. Yogyakarta, Indonesia. Physicochemical parameters during sampling included a relative humidity of 77% and an atmospheric pressure of 987–988 hPa.

*C. equisetifolia* leaves (5 kg) were crushed, ground, and homogenized with 5 L of ddH_2_O to obtain 2.5 L of leaf crude extract. The extract was filtered until the solution was clear yellow. Pro content was then measured and adjusted to 2.5 g mol^−1^ FW Pro as a stock crude extract (Bates et al., 1973). The stock was then diluted with ddH_2_O to three different Pro concentration levels: 50% (1.25 g mol^−1^ FW), 100% (2.5 g mol^−1^ FW), and a control without extract (0%). The leaf extract was stored overnight at 4°C to precipitate insoluble material in the filtrate and obtain a clear supernatant. The solution was mixed with a solution-grading [containing the active ingredients of polyacrylamide 75 g L^−1^ and polyvinyl acetate (PVAc) 75 g L^−1^] in a ratio of 1:1,000 (1 ml of solution-grading in 1 L of leaf extract). The extract was then applied to the abaxial surface of the rice leaf using a sprayer, with a total volume of 15 ml applied per block. Exogenous osmoprotectant was applied once when the plants had been treated with FTSW drought stress for 21 days (vegetative period).

### Relative gene expression analysis

Total RNA was isolated from 100 mg of young leaves per treatment using a FavorPrep Plant RNA Mini Kit 001-1 (Ping Tung Agricultural Biotechnology Park, Taiwan), after homogenization of the leaves using FARB buffer containing β-mercaptoethanol. The sample was incubated at room temperature for 5 min, followed by homogenization with 70% ethanol. The filtrate was separated by centrifugation, and the pellet was washed successively with wash buffers 1 and 2 containing 96% ethanol. RNA was dissolved in 50 μl of nuclease-free water and its purity was measured using a NanoDrop spectrophotometer. Before cDNA synthesis, RNA was further purified with a DNase I treatment kit (Sigma-Aldrich, Germany). cDNA was synthesized using an Excel RT Kit II (SMOBIO Technology, Taiwan). For gene expression analysis, the concentration of cDNA was adjusted to 500 ng µl^−1^. Primers for the targeted and reference genes are shown in **Table 2**.

**Table 2:** Targeted and reference gene primer sequences (Salsinha et al., 2022).

Accession number	Gene	Sequence (5′–3′)		bp	*T*_m_ (°C)
AF300970.1	*DREB1A*	F	ATCAAGCAGGAGATGAGCGG	134	59.4
		R	TGCCTCGTCTCCCTGAACTT		
KU159749.1	*DREB2A*	F	GGCTGAGATCCGTGAACCAA	120	58.3
		R	CGTGCTGTGGGACCATACAT		
AB028185.1	*OsNAC6*	F	TCATGGCCGGTGAACTTTGA	192	56.3
		R	GCACCATCTTTCTGCTGCTG		
AY870611.1	*OsWRKY45*	F	CGGCAGTGTAGTGTCAGTCA	128	58.3
		R	AGCTCCTTCCCCTTCTCCAT		
AY574031.1	*P5CS*	F	TGCGAGCAGGTTAAGGAACT	165	56.3
		R	TGCGAGCAGGTTAAGGAACT		
XM_015755303.2	*P5CR*	F	TGGAGTTGCTGCTGGTCTTC	116	56.3
		R	TATCCTTCAGCTGACCCGGA		
XM_015757226.2	*PRODH*	F	AGCAGAGGAGAACAGGGGAT	180	59.4
		R	TCGATCGCTTCACTCCCAAG		
EU650177	*Actin1*	F	AGCCACACTGTCCCCATCTA	155	59.4
		R	TCCCTCACAATTTCCCGCTC		

Gene expression analysis was performed following the Q-PCR Master Mix (SYBR, no ROX) protocol (SMOBIO Technology) with a quantitative real-time PCR machine (Bio-Rad CFX96) (Salsinha et al., 2022). The sample mix contained 1 µl of cDNA template, 1 µl of forward primer, 1 µl of reverse primer, 5 µl of 2 × -PCR Master Mix, and 2 µl of ddH_2_O. The PCR program consisted of a 2-min incubation at 95°C, followed by 39 cycles of 94°C denaturation for 30 s, annealing for 1 min at the *T*_m_ (melting temperature) of each targeted gene, and 72°C extension for 30 s, with subsequent storage at 4°C. A duplo–duplo experiment was used for the relative quantification of gene expression levels. Calculation of the relative expression level of each gene in each treatment followed the formula (Hong-zheng et al., 2017):

ΔCt unknown sample=Ct internal reference gene-Ct target gene

ΔCt calibrator=Ct reference gene in ref sample-Ct target gene in ref sample

ΔΔCt=ΔCt unknown sample-ΔCt calibrator

Relative expression=2^(-ΔΔCt)

### Physiological parameter measurement

#### Photosynthetic pigment measurement

Total chlorophyll and carotenoids were analyzed by the method of Harborne (1984). A total leaf sample of about 30 mg was homogenized with 80% cold acetone in the dark. The filtrate’s absorbance was measured at 470, 645, and 664 nm. Anthocyanins were measured by the Lotkowska method (Lotkowska et al., 2015). A leaf sample (250 mg) was ground with 1 ml of buffer containing 1-propanol, 37% HCl, and ddH_2_O. The sample was incubated at 95°C for 5 min and then at room temperature for 2 h. Absorbance was measured at 535, 620, and 720 nm.

#### Determination of Pro content

About 0.25 g of leaf samples was used to measure Pro content (Bates et al., 1973). The ground sample was homogenized with 3% sulfosalicylic acid solution (~5 ml) and filtered with Whatman No. 1 paper. Acetic and ninhydrin acid solutions were added to the sample at a ratio of 1:1:1. Samples were then incubated for 1 h at 94°C followed by ice-cooling. Two milliliters of toluene was added to the cold samples and mixed for 10 s. The absorbance of the colored phase (upper layer) was measured at 520 nm (GENESYS 10 UV Scanning, Thermo Fisher Scientific). Pro content was determined with a standard proline curve and calculated according to:


$$\text{Proline content }\left(x\right)\left({\text{mg g}}^{-1}\right)\frac{\frac{\text{Proline in cuvet }\left({\text{μg ml}}^{-1}\right)\times \text{V toluene }\left(\text{ml}\right)}{115.13\;\left({\text{μg μmol}}^{-1}\right)}}{\text{FW }\left(\text{g}\right)}$$

#### Enzymatic antioxidant measurement

Enzymatic antioxidant activity analysis for superoxide dismutase (SOD), ascorbate peroxidase (APX), and catalase (CAT) began with enzyme extraction following published protocols and measurements (Elevarthi and Martin, 2010). Approximately 0.2 g of ground leaves were homogenized with ~2 ml of 50 mM potassium phosphate buffer (pH 7.0) containing 1 mM EDTA and 1% PVP. The supernatant was kept below 4°C during measurement.

SOD activity was measured according to a previous protocol (Marklund and Marklund, 1974). The extract was prepared in a buffer containing 0.1 mM EDTA and Tris-HCl (pH 8.2) at room temperature. First, the absorbance of pyrogallol was measured as a control, followed by the sample with pyrogallol as blank. The enzymatic reaction was initiated by the addition of ddH_2_O and 4.5 mM pyrogallol. The absorbance was measured at 325 nm for 3 min. SOD activity was expressed as 50% pyrogallol autooxidation inhibition during incubation. Enzyme activity was calculated according to the formula:


$$\%\;\text{inhibition of pyrogallol autooxidation}=\frac{\;\Delta \text{test}}{\Delta \text{control}}x100\%$$

$$\left[\text{CuZn}\right]\text{ SOD activity }\left({\text{U L}}^{-1}\right)=\frac{\text{\% inhibition of pyrogallol autooxidation}}{50\%}$$

$$\text{SOD activity }\left({\text{U L}}^{-1}\right)=\left[\text{CuZn}\right]\text{ SOD activity}\times tV\;\left(\text{ml}\right)\times \frac{1}{V}XD$$

where Δtest = sample absorbance, Δ control = pyrogallol absorbance, tV (ml) = total assay volume (buffer + ddH_2_O + pyrogallol + enzyme sample), *V* (ml) = volume of enzyme sample, and *D* = dilution factor.

APX activity was measured by mixing 100 µl of enzyme extract with a buffer containing 0.05 mM sodium phosphate, 0.1 mM EDTA, 0.05 mM ascorbic acid, and ddH_2_O (Elevarthi and Martin, 2010). The enzymatic reaction was started by adding 0.8 ml of 3% (v/v) hydrogen peroxide (H_2_O_2_) solution, and the absorbance was measured for 3 min at 290 nm and calculated according to:


$$\text{APX activity }\left({\text{U L}}^{-1}\right)=\frac{\left|\Delta Abs\right|\times tV}{\Delta t\times \epsilon \times L\times V}$$

where Δ*Abs* = absorbance change during *t* (min), *tV* (ml) = total assay volume (buffer reagent + enzyme sample), Δ*t* = time (3 min), ϵ extinction coefficient (2.8 mM^−1^ cm^−1^), *V* (ml) = volume of enzyme sample, *L* = cuvet diameter (1 cm).

CAT activity was measured according to a published protocol (Elevarthi and Martin, 2010) by reacting 200 µl of the enzyme extract with a buffer containing 50 mM sodium phosphate (pH 7.0). A solution of 3% H_2_O_2_ was added to start the reaction, followed by incubation at room temperature for 1 min. The absorbance was measured at 240 nm for 3 min and CAT activity was expressed as the decrease in H_2_O_2_ per minute per mg protein:


$$\text{CAT activity }\left({\text{mmol H}}_{2}{\text{O}}_{2}{\text{ min}}^{-1}{\text{ g}}^{-1}\text{ FW}\right)=\frac{\left|\Delta Abs\right|\times tV}{\Delta t\times \epsilon \times L\times \;FW}$$

where Δ*Abs* = absorbance change during *t*, *tV* (ml) = total assay volume (buffer reagent + enzyme sample), Δ*t* = time (3 min), ϵ = extinction coefficient (40 mM^−1^ cm^−1^), *L* = cuvet diameter (1 cm), FW = fresh weight (g).

The H_2_O_2_ content was measured using the method of Bouazizi et al. (2007). Leaf samples (0.25 g) were ground with 0.1% TCA. The supernatant (0.5 ml) was reacted with 10 mM ferrous ammonium sulfate, 2.5 M potassium thiocyanate, and 50% TCA, followed by incubation at room temperature for 1 min. The absorbance was measured at 390 nm. The hydrogen peroxide content was determined by comparing it with the standard H_2_O_2_ curve.


$${\text{H}}_{2}{\text{O}}_{2}\text{ content}=\frac{1,000\times \left(A532-A600\right)}{155}X\frac{1}{FW}XD$$

where *FW* = fresh weight (g) and *D* = dilution factor.

### Morphological parameter analysis

The plant morphological parameters measured included plant height, number of tillers, number of leaves, and root length measured at 49 DAPs. Plant height was measured from the uppermost leaf to the base of the stem at the soil surface. The number of tillers was counted from the base above the soil surface that showed growth of more than two leaves. The total number of leaves measured was the leaves that had opened on each tiller. The longest root length was measured from the base near the soil surface to the longest root tip. The fresh weight of roots and shoots was measured separately using analytical scales. Each shoot and root part were then stored in an oven at 65°C until the weight became constant. The dry weight of each root and shoot was then measured.

### Statistical analysis

The data obtained were analyzed for significant differences between treatments using a multivariate two-way ANOVA. Differences between groups were analyzed using Duncan’s multiple range test at a 95% confidence interval. Correlation between parameters was determined with Pearson’s *r* correlation analysis using SPSS (IBM-SPSS Ver 25.00.US). Data visualization was performed using GraphPad Prism 9.3.1.

## Results

### Expression of transcription factor and proline-related genes

In this study, osmoprotectants derived from the halophyte plant C. *equisetifolia* were extracted. From the test results that have been carried out, it can be observed at the expression level of TFs and genes related to the proline synthesis pathway as well as other physiological processes downstream of Pro accumulation.

**Figure 1:**
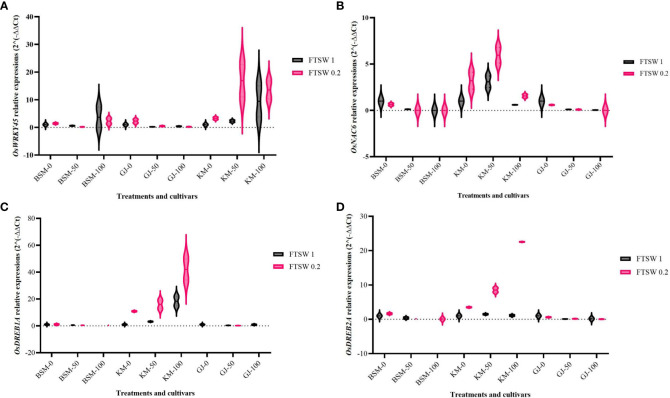
Expression level of **(A)** *OsWRKY*, **(B)** *OsNAC6* **(C)** *OsDREB1A*, and **(D)** *OsDREB2A* in *Oryza sativa* leaves after treatment with two levels of FTSW (fraction of transpirable soil water), FTSW 1 (control) and FTSW 0.2 (drought stress), and three levels of exogenous osmoprotectant application, 0% (low), 50% (moderate), and 100% (high), in the cultivars BSM, KM, and GJ. A duplo–duplo experiment with three replications each was used to express the relative quantification of gene expression levels with three replications of each treatment group.

**Figure 1** shows the expression levels of drought-responsive transcription factors (*OsWRKY45*, *OsNAC6*, *OsDREB1A*, and *OsDREB2A*) as a result of drought stress treatment and exogenously applied osmoprotectant in the local rice cultivars BSM, GJ, and KM. **Figure 1A** shows that in the BSM cultivars, exposure to exogenous osmoprotectants at low and medium concentrations did not significantly increase *OsWRKY45* expression in GJ cultivars, while in KM cultivars, an increase in the concentration of exogenous osmoprotectants led to an increased level of *OsWRKY45* expressions, with the highest level shown in plants under drought stress.

High levels of *OsNAC6* expression was found in BSM and GJ cultivars treated with a lower concentration of osmoprotectants (**Figure 1B**). The gene’s expression in BSM and GJ decreased as the concentration of osmoprotectants increased. KM cultivars showed an increase in the expression level of *OsNAC6* under drought stress and moderate osmoprotectants. The application of high concentrations of osmoprotectants caused a decrease in the expression level of *OsNAC6* in KM.

**Figure 1C** shows the change in the expression level of *OsDREB1A*. KM cultivars showed an increase in the expression level of *OsDREB1A* in line with the increasing concentration of osmoprotectants under drought stress. The highest expression level of *OsDREB1A* was observed in KM plants subjected to drought stress. **Figure 1D** shows an increasing expression level of *OsDREB2A* in KM cultivar with the increase in the concentration of osmoprotectants but not with 100% osmoprotectant. In BSM and GJ, neither drought stress nor the application of osmoprotectant resulted in significant changes in the expression level of *OsDREB2A*.

The expression of the genes encoding TFs *OsWRKY45, OsNAC6, OsDREB1A*, and *OsDREB2A* is induced by drought stress independently whether osmoprotectants are applied or not. The application of exogenous osmoprotectants mainly increased the expression levels of these TFs in the KM cultivar under drought conditions. Furthermore, TFs play a role in activating gene expression in the proline metabolic pathway (Zhang et al., 2013).

Pro metabolism involves the activity of several functional genes. Increased proline accumulation requires activation of several genes involved in Pro synthesis—*OsP5CS* encodes P5CS (Δ 1-Pyrroline-5-carboxylate synthetase), while *OsP5CR* encodes P5CR (Δ 1-Pyrroline-5-carboxylate reductase), which functions in the proline synthesis pathway *via* glutamate (Kavi Kishor et al., 2005; Salsinha et al., 2022). Proline catabolism is carried out by PRODH (proline dehydrogenase), which is encoded by the *OsProDH* (Luo et al., 2020; Salsinha et al., 2022). We examined the transcript accumulation of the *OsP5CS1, OsP5CR*, and *OsProDH* genes in the rice cultivars under control and drought stress condition in combination with supplied osmoprotectants.

**Figure 2:**
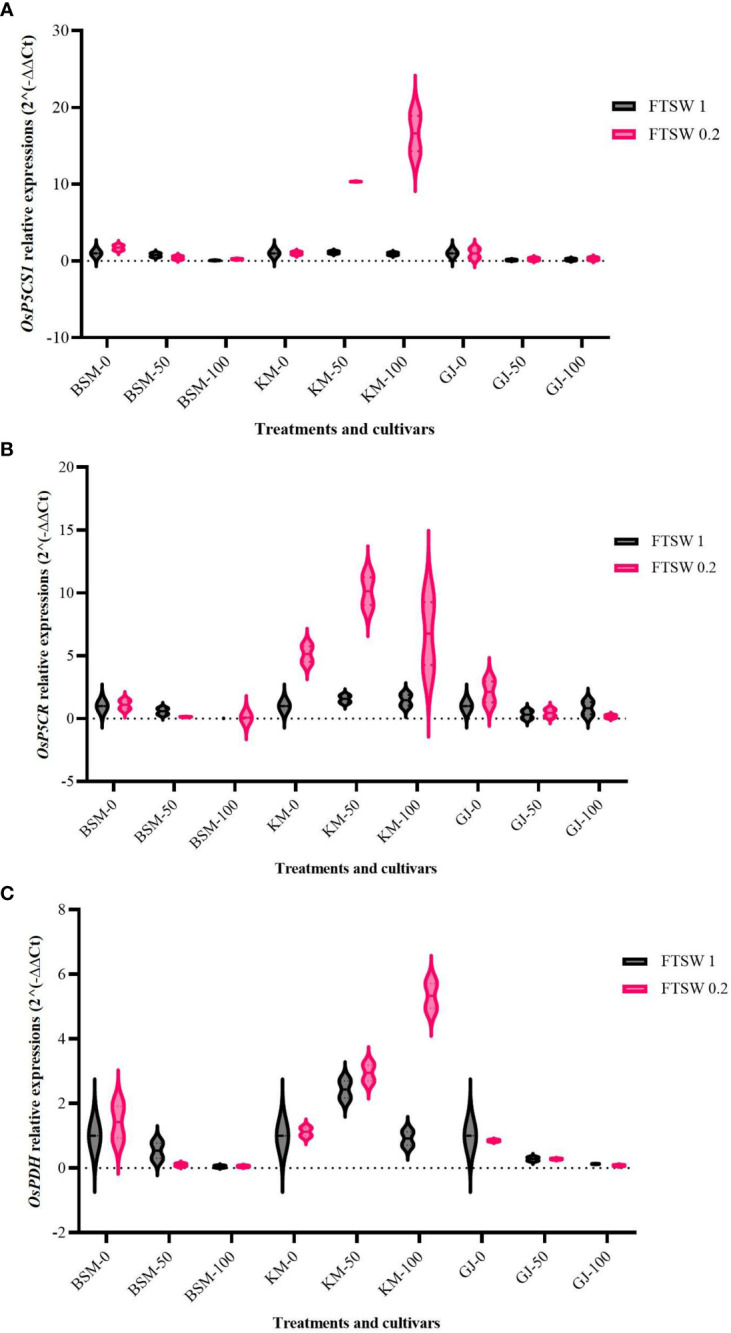
Expression levels of **(A)** *OsP5CS1*, **(B)** *OsP5CR*, and **(C)** *OsProDH*, in the three cultivars *BSM*, *KM*, and *GJ* grown at one of the two levels of FTSW and treated with three different concentrations of exogenous osmoprotectant. A duplo–duplo experiment with three replications each was used to express the relative quantification of gene expression levels with three replications of each treatment group.

The expression levels of *OsP5C1* increased only in KM treated with osmoprotectant during drought stress (**Figure 2A**). Analysis of variance showed that the expression of *OsP5C1* was significantly different (*p*< 0.05) between KM cultivars and other cultivars. The *OsP5CR* expression of KM during drought stress was elevated in comparison to the control conditions and in comparison to the other cultivars during drought (**Figure 2B**). Drought stress combined with 50% exogenous osmoprotectant in KM showed the highest *OsP5CR* expression level (more than ninefold). The expression of the *OsProDH* gene in KM cultivar increases in both drought stress and 100% applied osmoprotectant (**Figure 2C**). Under control conditions, KM showed decreased expression of *OsProDH* when exposed to high concentrations of osmoprotectants.

### Pro accumulation

**Figure 3:**
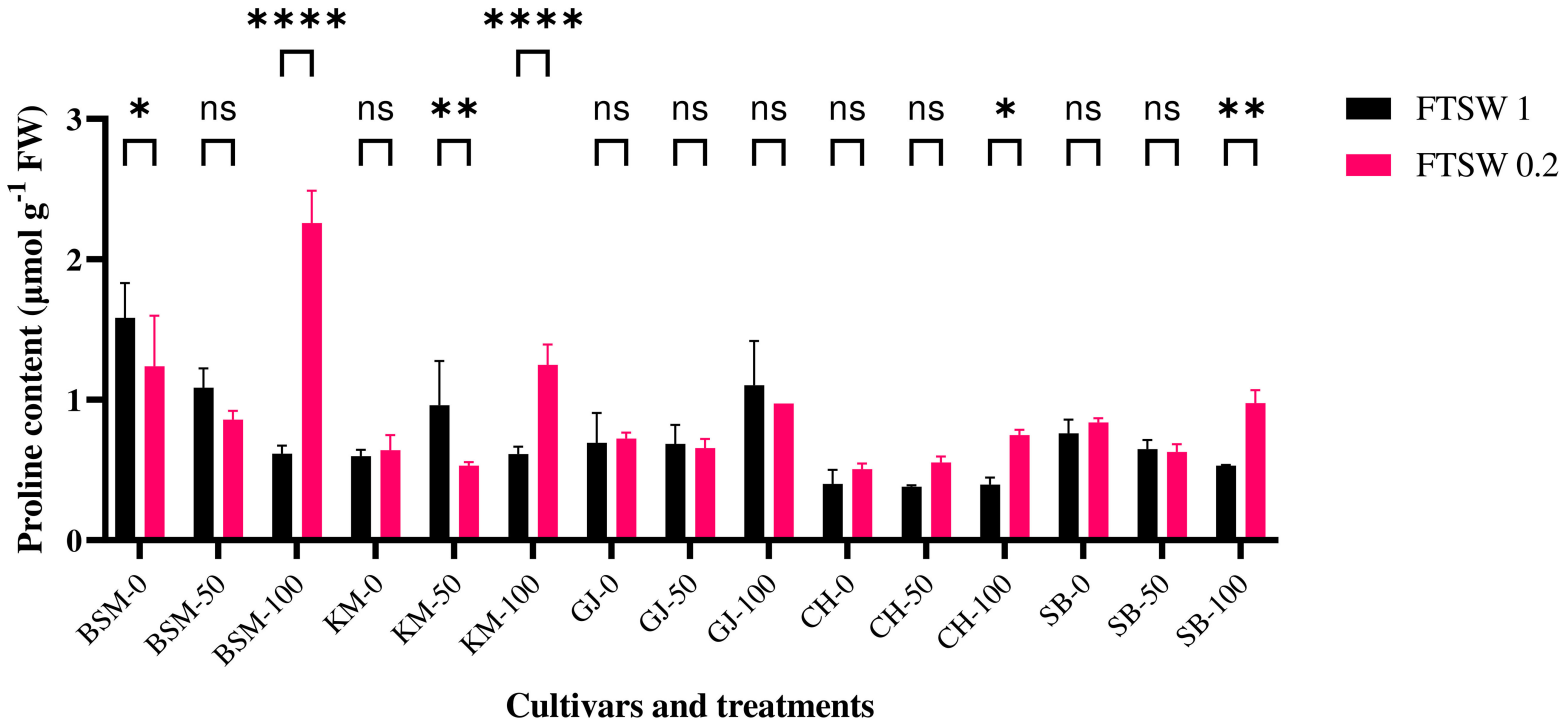
Differences in Pro levels at the end of drought stress treatment with FTSW 1 (control) and FTSW 0.2 (drought stress) in rice cultivars GJ, KM, BSM, CH (drought-susceptible control), and SB (drought-tolerant control) exposed to osmoprotectant concentrations of 0% (low), 50% (moderate), and 100% (high). ns=no significant difference with p value>0.05, * p value < 0.05, ** p value=0.02, **** p value < 0.0001 based on 95% confidence level in ANOVA two-way test with three replications each.

Pro accumulation is induced by several processes during drought stress. In this study, proline contents were measured in plants after drought stress treatment and application of osmoprotectants. In this phase, the plants showed significant differences in proline accumulation (*p*< 0.05) in response to the different treatments (**Figure 3**). BSM cultivars exposed to drought stress were detected to have higher proline levels than the other cultivars at low concentrations of osmoprotectant exposure. The application of high amount of osmoprotectant under drought gives a significant difference of proline accumulation in BSM cultivars. In high concentrations of osmoprotectant treatment, BSM cultivars also showed the highest proline levels.

#### Oxidative responses of plants

**Figure 4:**
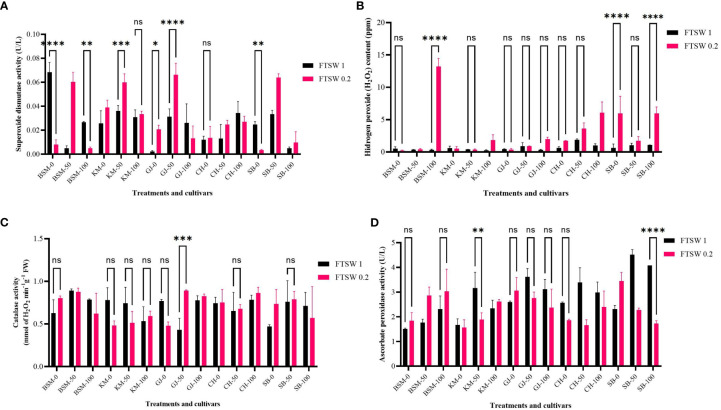
Enzymatic antioxidant activity of **(A)** SOD, **(B)** the product of SOD catalysis in the form of H_2_O_2_, and the catalytic activity of **(C)** APX and **(D)** CAT at the end of the drought stress treatment indicated by FTSW 0.2 and control (FTSW 1) in the rice cultivars GJ, KM, BSM, CH (drought-susceptible control), and SB (drought-tolerant control). The cultivars were exposed to osmoprotectant concentrations of 0% (low), 50% (moderate), and 100% (high). ns=no significant difference with p value>0.05, * p value < 0.05, ** p value=0.02, *** p value= 0.0001, **** p value < 0.0001 based on 95% confidence level in ANOVA two-way test with three replications each.

Drought stress affects changes in the physiological characters of rice plants. At the same time, the application of osmoprotectants also has an impact on plant resistance to drought. Changes in the oxidative stress response of plants as a result of drought stress and osmoprotectant treatment are depicted in **Figure 4**. On exposure to low concentrations of osmoprotectants, BSM and SB cultivars showed a significantly different decrease in SOD activity (*p*< 0.05), while other cultivars showed an increase in activity in the exposure to the 50% osmoprotectant treatments.

**Figure 4B** shows the different levels of H_2_O_2_ content. Exposure to low concentrations of osmoprotectants did not give a significant effect (*p* > 0.05) on H_2_O_2_ level changes in most of the cultivars. Non-significant differences (*p* > 0.05) were found in all cultivars on exposure to 50% osmoprotectant treatments. Exposure to high concentrations of osmoprotectants caused a substantial increase in H_2_O_2_ levels in BSM, CH, and SB.

ROS scavenging activity is also carried out by enzymes such as CAT and APX in all major H_2_O_2_ production compartments in the internal cell environment such as peroxisomes, mitochondria, cytosol, and chloroplast (You et al., 2019). **Figure 4C** significantly shows differences in APX activity among rice cultivars under drought stress and after osmoprotectant application. When exposed to 50% osmoprotectant, most cultivars showed significant differences (*p*< 0.05) between control and severe drought stress. The SB cultivars showed significant differences (*p*< 0.05) when exposed to high concentrations of osmoprotectants, while the other cultivars showed no significant differences between control and drought stress conditions.

CAT activity in Indonesia’s local rice exposed to drought stress and osmoprotectant application is shown in **Figure 4D**. On exposure to low concentrations of osmoprotectants, GJ and BSM showed a decrease in CAT activity. The BSM, CH, and SB cultivars showed a non-significant increase of CAT activity (*p*< 0.05) between control and drought stress. GJ plants on moderate osmoprotectant exposure displayed a significant increase (*p*< 0.05) in CAT activity. At 100% concentrations of osmoprotectants, there was no significant difference (*p* > 0.05) between control and drought stress conditions in any cultivar. Changes in enzymatic antioxidant activity affect the physiological performance of plants, which is reflected in changes in photosynthetic pigments.

#### Photosynthetic responses

**Figure 5:**
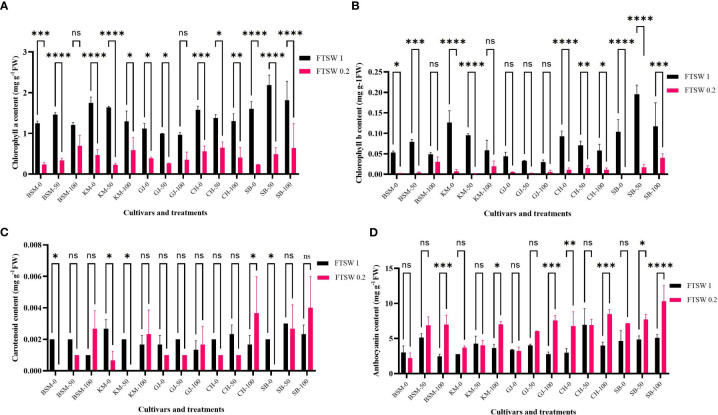
Differences in levels of photosynthetic pigments **(A)** chlorophyll a, **(B)** chlorophyll b, **(C)** carotenoids, and antioxidant pigment **(D)** anthocyanins of the rice cultivars GJ, KM, BSM, CH (drought-susceptible control), and SB (drought-tolerant control) at the end of the drought stress treatment with FTSW 1 (control) and FTSW 0.2 (drought stress). The cultivars were exposed to osmoprotectant concentrations of 0% (low), 50% (moderate), and 100% (high). Ns, no significant difference, *p< 0.05 based on 95% confident level in ANOVA two-way test with 3 replications each.

The photosynthetic response of plants to environmental conditions correlates with the content of photosynthetic pigments. **Figure 5** shows the content of the photosynthetic pigments chlorophyll a, chlorophyll b, carotenoids, and anthocyanins as an antioxidant compound. Chlorophyll a (**Figure 5**) generally decreased when plants were exposed to drought conditions in all cultivars. The deeper the loss of chlorophyll content between control and drought conditions, the stronger the susceptibility of a cultivar to drought stress.

**Figure 5** shows the differences in chlorophyll b between control and drought stress conditions in some cultivars exposed to osmoprotectants. In all cultivars, exposure to low concentrations of osmoprotectants caused a decrease in chlorophyll b level under drought stress. Under 100% concentrations of osmoprotectants, some cultivars showed changes in chlorophyll b level between control and drought conditions. All cultivars under the control condition showed a gradual decrease of chlorophyll b content except for SB, in which the content was higher concentration at the 100% concentration of exogenous osmoprotectant.

In addition to chlorophyll a and b, the levels of carotenoids also changed when plants were exposed to drought conditions and when the osmoprotectant was applied. On exposure to low concentrations of osmoprotectants (**Figure 5**), KM, BSM, and SB cultivars showed significant differences (*p*< 0.05) between control and drought stress conditions. CH showed a significant difference (*p*< 0.05) in carotenoid levels between control and drought stress conditions on exposure to 100% concentrations of osmoprotectants, while KM with 50% osmoprotectant also showed a significant difference (*p*< 0.05).

Anthocyanin is a plant pigment that plays a role in ROS scavenging. Anthocyanin levels (**Figure 5**) increased in cultivars exposed to drought stress and osmoprotectants. Exposure to osmoprotectants at low and 50% concentration levels did not affect anthocyanin levels of BSM, GJ, and KM cultivars under drought compared to control, except for CH. On exposure to high concentrations of osmoprotectants, cultivars GJ, KM, BSM, CH, and SB experienced an increase in anthocyanin levels relative to drought stress compared to control.

### Morphological changes

**Figure 6:**
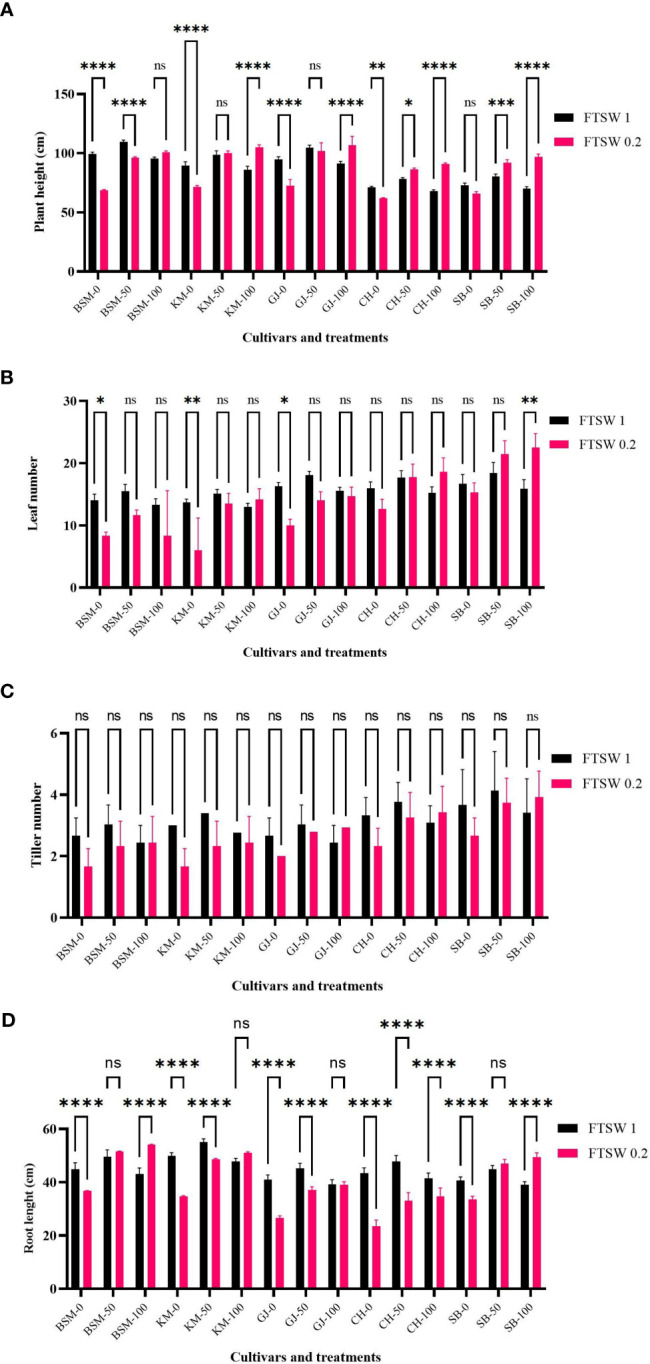
Growth parameters of **(A)** plant height, **(B)** leaf number, **(C)** tiller number, and **(D)** root length of the drought stress treatment rice cultivars GJ, KM, BSM, CH (drought-susceptible control), and SB (drought-tolerant control) at the end of the drought stress treatment with FTSW 1 (control) and FTSW 0.2 (drought stress). The cultivars were exposed to osmoprotectant concentrations of 0% (low), 50% (moderate), and 100% (high). Ns, no significant difference with p value>0.05, * p value< 0.05, ** p value=0.02, *** p value= 0.0001, **** p value < 0.0001 based on 95% confident level in ANOVA two-way test with three replications each.

Morphological characters are the result of the accumulation of plant physiological processes over time. Several studies have shown that root morphological characters play an important role in adaptation to drought (e.g., Liu et al., 2017). Plants with good adaptation mechanisms have high root biomass and dense root systems. Under drought stress conditions (**Figure 6**), KM as a drought-susceptible cultivar showed increased tolerance when exogenous osmoprotectant was applied with elevated plant height, number of leaves, and root length. In contrast, the number of tillers did not change in any cultivar when exposed to drought stress conditions and subjected to exogenous osmoprotectants at various concentrations (*p* > 0.05). Without exogenous osmoprotectants, KM showed a significant decrease (*p*< 0.05) in plant height, number of leaves, and total root length. BSM and SB showed similar patterns but with a non-significant decrease between drought stress and control conditions when no exogenous osmoprotectant was applied.

**Figure 7:**
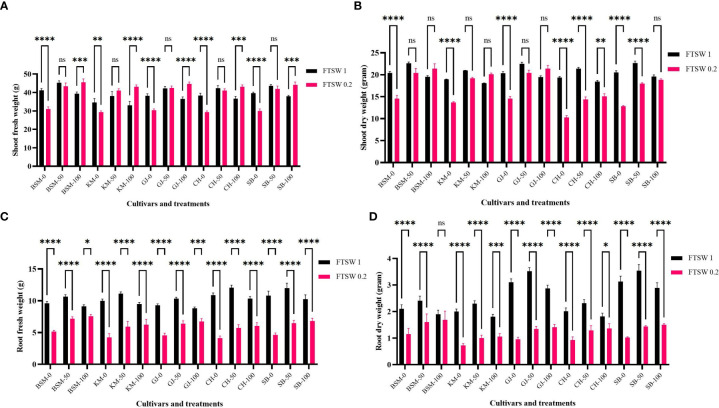
The biomass parameters of **(A)** root fresh weight, **(B)** root dry weight, **(C)** shoot fresh weight, and **(D)** shoot dry weight after drought stress treatment with FTSW 1 (control) and FTSW 0.2 (severe drought stress) in rice cultivars GJ, KM, BSM, CH (drought-susceptible control), and SB (drought-tolerant control) at the end of the drought stress treatment with FTSW 1 (control) and FTSW 0.2 (drought stress). The cultivars were exposed to osmoprotectant concentrations of 0% (low), 50% (moderate), and 100% (high). Ns, no significant difference, *p< 0.05 based on 95% confident level in ANOVA two-way test with three replications each. ns=no significant difference with p value>0.05, * p value < 0.05, ** p value=0.02, *** p value= 0.0001, **** p value < 0.0001 based on 95% confidence level in ANOVA two-way test with three replications each.

**Figure 7** shows changes in plant biomass when treated with drought stress and exogenous osmoprotectant treatment. Overall, in conditions without exogenous osmoprotectants (0%), plants showed a decrease in root fresh weight, root dry weight, and shoot fresh weight and dry weight (**Figures 7A–D**, respectively) between control and stress treatment. A significant decrease shown by KM and CH. Several cultivars such as BSM and GJ did not show significant changes in biomass (*p* > 0.05) under drought stress compared with controls even though they were exposed to moderate and high concentrations of exogenous osmoprotectants.

### Pearson’s *r* correlation analysis

**Figure 8:**
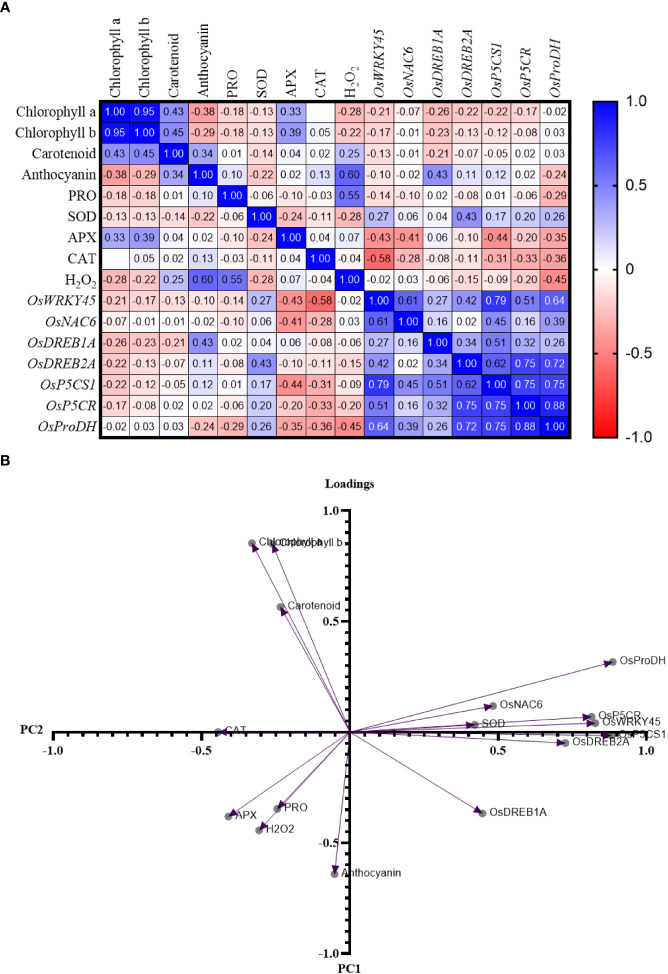
**(A)** Pearson’s *r* correlation matrix and **(B)** principal component analysis of parameters in rice cultivars GJ, KM, BSM, CH (drought-susceptible control), and SB (drought-tolerant control) at the end of the drought stress treatment with FTSW 1 (control) and FTSW 0.2 (drought stress). The cultivars were exposed to osmoprotectant concentrations of 0% (low), 50% (moderate), and 100% (high).

A positive value indicates a positive correlation between the two parameters being compared, meaning that an increase in the value of one parameter is accompanied by an increase in the other parameter. Positive correlations were found in comparisons between expression level of TFs (*OsWRKY45*, *OsNAC6*, *OsDREB1A*, and *OsDREB2A*) and Pro-related genes (*OsP5CS1, OsP5CR*, and *OsProDH*) (**Figure 8A**). Mostly, the gene expression level and the activity of APX and CAT were found negatively correlated.

The results of the principal component analysis revealed a grouping based on Eigenvalue (**Figure 8B**). Based on the analysis with principal component (PC) I, the characters *OsNAC6*, *OsProDH*, *OsP5CR*, *OsDREB1A*, *OsDREB2A*, *OsWRKY45*, and *OsP5CS*1 were found to contrast with the characters of chlorophyll a, chlorophyll b, carotenoids, Pro, anthocyanins, H_2_O_2_, APX, CAT, and SOD. Based on PC II, the characters of chlorophyll A, B, carotenoids, SOD, *OsProDH*, *OsNAC6*, *OsP5CR*, and *OsWRKY45*, and the expression of *OsP5CS*1 contrast with the character of the activity of APX, H_2_O_2_, Pro, anthocyanin, and CAT, and the expression of *OsDREB1A*.

## Discussion

One of the widely known osmolytes that act as metabolic markers under abiotic stress conditions is Pro. The accumulation of Pro in plants is closely related to its function in osmotic adjustment. This process allows the plant to conserve water by reducing the rate of water loss from subcellular compartments to the extracellular environment (Farahani et al., 2013). In rice, the increased accumulation of Pro is proportional to the level of drought stress (Raye et al., 2018). In stress-susceptible plants (KM), the addition of exogenous osmoprotectants acts as a trigger that plays a role in the activation of TFs and Pro metabolism regulatory genes, while in drought-tolerant cultivars (BSM and GJ), the addition of exogenous osmoprotectants acts as a negative regulatory signal to stop endogenous proline biosynthesis in cells.

In this study, an analysis of TFs and the expression levels of genes in Pro synthesis pathways and their relation to physiological characteristics related to Pro accumulation has been carried out. The TFs involved in the Pro regulatory pathways are *OsDREB1A, OsDREB2A, OsNAC6*, and *OsWRKY45*. These TFs provide molecular switches for gene expression in the response to drought stress. In this study, the drought stress response was observed through the expression level of targeted genes in the Pro synthesis pathway including *OsP5CS1, OsP5CR*, and *OsProDH*.

**Figure 9:**
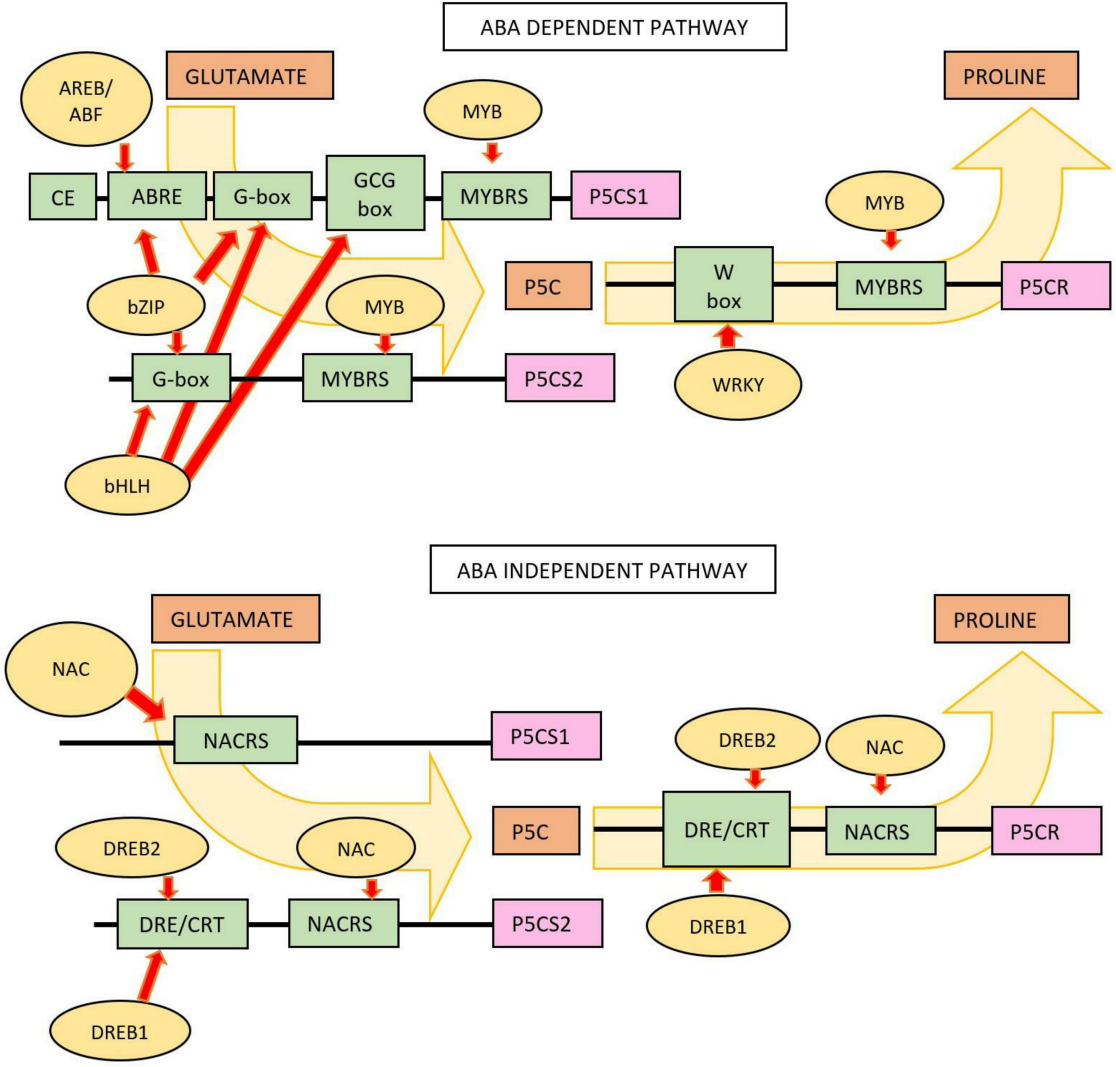
Pro synthesis pathways involving TFs and genes in the process of proline anabolism. Regulation of Pro accumulation by an ABA-dependent pathway. TFs included in this pathway are *AREB/ABF* (ABA-responsive element binding protein/ABRE-binding factor), bZIP (basic region, leucine zipper), *bHLH* (basic helix–loop–helix), MYB (v-Myb myeloblastosis viral oncogene homolog), and *WKRY* (a 60-amino-acid region that is defined by the conserved amino acid sequence WRKYGQK at its N-terminal end). Regulation of Pro accumulation by an ABA-independent pathway. The TFs involved are the *NAC* (no apical meristem—Petunia, *ATAF1–2*—Arabidopsis thaliana activating factor, and *CUC2—*cup-shaped cotyledon, Arabidopsis) and *DREB1A* and *DREB2A* (dehydration responsive element-binding) groups.

Based on **Figure 9**, there are two Pro regulatory pathways, namely, ABA-dependent and ABA-independent pathways. In the ABA-dependent pathway, Pro accumulation is regulated by the *AREB/ABF, bZIP, bHLH*, and *WKRY* TFs group. These TFs activate MYB in glutamate catalysis as a Pro precursor to P5C. In the reduction step of P5C to Pro, the TFs involved is a member of the *WRKY* family, which regulates the synthesis of the P5CR enzyme. In the ABA-independent pathway, proline accumulation is regulated by the *AREB/ABF* and *NAC* TFs that are involved in activating P5CS1 and P5CS2 synthesis and P5C anabolism through the catalytic activity of P5CR.

In this study, the drought-tolerant cultivars (BSM and GJ) did not show a significant increase in the expression level of *OsWRKY45*, while in the susceptible KM cultivar, an increase in the concentration of exogenous osmoprotectants also led to an increase in the expression level of *OsWRKY45*, with the highest levels shown in plants under drought stress.

The expressions level of *OsNAC6* was found to be higher in the KM when treated with drought and osmoprotectant at the concentration level of 50%. In drought-tolerant cultivars (BSM and GJ), there was no significant differences in gene expressions at any level of exogenous osmoprotectant. Moreover, *OsNAC6* expression decreased as the concentration of osmoprotectants increased. Previous studies (Nakashima et al., 2007; Parida et al., 2016; Karim et al., 2021) showed that *NAC* plays a role in growth and development, fluctuations in hormones, leaf senescence, and plant defense mechanisms against environmental stresses. This gene is induced by stress. Plants with higher *NAC* expression levels tend to have the potential to survive abiotic stresses (Baillo et al., 2019).

Under drought stress, the expression level of *OsDREB1A* in BSM and GJ rice decreased as the exogenous osmoprotectant concentration increased from 0% to 50%, but did not differ between 50% and 100% concentrations. The susceptible KM cultivar showed an increased level of *OsDREB1A* with 100% exogenous osmoprotectant application.

During drought stress, the expression level of *OsDREB2A* in drought-tolerant cultivars (BSM and GJ) increased at a 50% concentration of exogenous osmoprotectant but then decreased as the concentration rose to 100%. In drought-tolerant cultivars as above, an increase in the exogenous osmoprotectant concentration caused a decrease in the expression level of *OsDREB2A*. This increase in *DREB* TF activity at high concentrations of exogenous osmoprotectant treatment theoretically increases the transcription of *P5CS1* and *P5CS2* in the ABA-dependent pathway, as well as in the ABA-independent pathway through NAC activation. This whole process leads to an increase in endogenous Pro synthesis and its accumulation.

Pro is synthesized when plants experience drought. The mechanism of Pro biosynthesis can differ between species and even between cultivars. Previous research (Vendruscoloa et al., 2007) on *Zea mays* and *Triticum aestivum* found that Pro accumulation in plants is a defense mechanism through osmotic adjustment to prevent cell damage due to dehydration and the ROS scavenging mechanism in the oxidative response pathway. Our data show the expression of *OsP5CS* in NTT local rice leaves treated with drought and exogenous osmoprotectant. The expression levels of *OsP5CS1* in KM with drought stress and control treatments increased when exposed to osmoprotectants at 50% and 100%.

In drought-tolerant cultivars (BSM, GJ, and SB), the application of 50% and 100% of osmoprotectant decreased the expression levels of *OsP5CS1* in both the drought stress and the control conditions. In susceptible cultivars (KM), drought stress gave a significant increase in this gene expression at the osmoprotectant levels of 50% and 100%. Based on this study, KM showed increased expression levels of *OsP5CS* and *OsP5CR* with increasing exogenous osmoprotectant concentration, while GJ and BSM showed the opposite expression.

The same pattern was also found in the expression of *OsProDH*, which catalyzes the reduction of excess Pro into P5CS. In drought-tolerant cultivars, *OsProDH* expression increased as Pro accumulation levels increased, while in the drought-susceptible cultivar (KM), the opposite occurred. This may have happened because the accumulation of exogenous Pro became a feedback signal to reduce high *OsProDH* activity in drought-tolerant rice under drought. The expression of *OsProDH1* and *OsProDH2* increased during the senescence and recovery phase (Guo et al., 2020). This upregulation functions to reduce the level of toxicity of Pro/P5C.

Each cultivar accumulates Pro with different strategies. In control conditions without osmoprotectant, the drought-tolerant cultivar GJ showed the highest Pro content when treated with a high concentration of osmoprotectant. During drought, GJ and KM (drought susceptible) cultivars tend to show no significant differences between osmoprotectant level, while BSM (drought-tolerant) shows higher Pro accumulation with the application of osmoprotectant.

A study by Hossain et al, 2014 ; Shemi et al., 2021) has shown that exogenous Pro increases the antioxidant activity of plants against abiotic stress. This finding is in agreement with another report (Nounjan et al., 2012) that showed an increase in APX, POX, CAT, and APX activities under stress conditions and a faster recovery period with the application of exogenous Pro.

**Figure 10:**
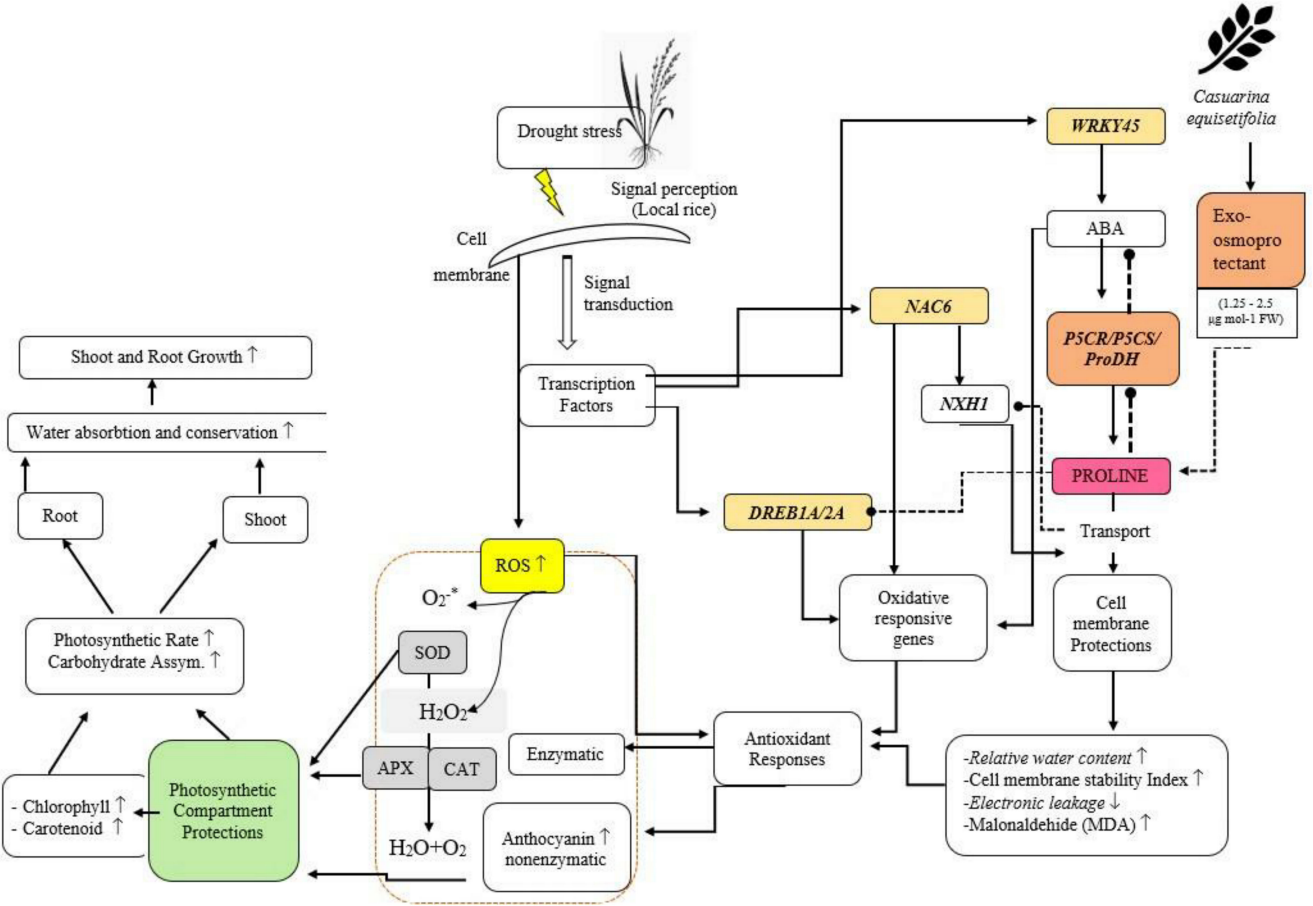
Schematic diagram of Pro regulation and physiological changes under drought stress in NTT local rice treated with exogenous osmoprotectants (containing 1.25–2.5 µg mol^−1^ FW). The image shows the interaction between drought stress treatment and physiological changes and the foliar application of exogenous osmoprotectants in NTT, Indonesia local rice plants.

Foliar application of exogenous osmoprotectants derived from *C.equisetifolia* leaf extract causes accumulation of osmoprotectants in drought-tolerant plant organs. This accumulation leads to an inhibitory effect of *OsP5CS* and *OsP5CR* expression in drought-tolerant cultivars but instead activates the expression of *OsP5CS* and *OsP5CR* on drought-susceptible cultivars. Differences in the regulation of the two groups are presented in **Figure 10**. *OsProDH* gene expression increased in tolerant plants, but decreased in drought-susceptible plants (**Figure 10**). A decreased *OsProDH* expression level of drought-susceptible plants resulted in an accumulation of proline which induced cell membrane protection, which was observed through an increase in relative water content and higher cell membrane stability index.

In drought-tolerant plants, there was no apparent difference in the level of Pro accumulation or SOD activity after the application of exogenous osmoprotectants. The increased protection of cell compartments in drought-susceptible cultivars led to a decrease in ROS in cells, which, in turn, led to an increase in physiological activities such as photosynthesis to support the formation of cell biomass.

Based on the present research, the regulatory pathway for exogenous osmoprotectants in Indonesia’s local upland rice involves several important components at the molecular, cellular, tissue, and organ level. In general, under normal conditions without exogenous osmoprotectant treatment, plants can be divided into groups that are tolerant and susceptible to drought. After the cultivars were given exogenous osmoprotectants, there were differences in the response to drought stress (tolerant and susceptible) and osmoprotectants applied (responsive and non-responsive).

## Conclusion

Foliar application of exogenous osmoprotectants derived from *C.equisetifolia* caused accumulation of proline in drought-susceptible plants. The existence of these extracts stabilizes leaf cells and supports photosynthetic compartments and carbon assimilation in plants, leading to growth.

## Data availability statement

The original contributions presented in the study are included in the article/**Supplementary Material**. Further inquiries can be directed to the corresponding author.

## Author contributions

Conceptualization: YS, DR, and YP. Data curation: DSR and TA. Formal analysis: DR and DI. Funding acquisition: YP. Methodology: YS, DI, and YP. Software: YS, DSR, and TA. Validation: DR, DI, and TA. Visualization: YS and DSR. Writing—original draft: YS, DR, DI, YP, and DSR. Writing—review and editing: DR, TA, and YP. All authors contributed to the article and approved the submitted version.
